# Effect of glucose on poly-γ-glutamic acid metabolism in *Bacillus licheniformis*

**DOI:** 10.1186/s12934-017-0642-8

**Published:** 2017-02-08

**Authors:** Wencheng Yu, Zhen Chen, Hong Ye, Peize Liu, Zhipeng Li, Yuanpeng Wang, Qingbiao Li, Shan Yan, Chuan-jian Zhong, Ning He

**Affiliations:** 1 Department of Chemical and Biochemical Engineering, College of Chemistry and Chemical Engineering, Xiamen University, Xiamen, 361005 People’s Republic of China; 2 The Key Lab for Synthetic Biotechnology of Xiamen City, Xiamen University, Xiamen, 361005 People’s Republic of China; 3 Fujian Provincial Key Laboratory of Fire Retardant Materials, College of Materials, Xiamen University, Xiamen, 361005 People’s Republic of China; 4 Department of Chemistry, State University of New York at Binghamton, Binghamton, NY 13902 USA

**Keywords:** Glucose, γ-PGA, Polysaccharide, Carbon control protein, *B. licheniformis*

## Abstract

**Background:**

Poly-gamma-glutamic acid (γ-PGA) is a promising macromolecule with potential as a replacement for chemosynthetic polymers. γ-PGA can be produced by many microorganisms, including *Bacillus* species. *Bacillus licheniformis* CGMCC2876 secretes γ-PGA when using glycerol and trisodium citrate as its optimal carbon sources and secretes polysaccharides when using glucose as the sole carbon source. To better understand the metabolic mechanism underlying the secretion of polymeric substances, SWATH was applied to investigate the effect of glucose on the production of polysaccharides and γ-PGA at the proteome level.

**Results:**

The addition of glucose at 5 or 10 g/L of glucose decreased the γ-PGA concentration by 31.54 or 61.62%, respectively, whereas the polysaccharide concentration increased from 5.2 to 43.47%. Several proteins playing related roles in γ-PGA and polysaccharide synthesis were identified using the SWATH acquisition LC–MS/MS method. *CcpA* and *CcpN* co-enhanced glycolysis and suppressed carbon flux into the TCA cycle, consequently slowing glutamic acid synthesis. On the other hand, *CcpN* cut off the carbon flux from glycerol metabolism and further reduced γ-PGA production. *CcpA* activated a series of operons (*glm* and *epsA*-*O*) to reallocate the carbon flux to polysaccharide synthesis when glucose was present. The production of γ-PGA was influenced by *NrgB*, which converted the major nitrogen metabolic flux between NH_4_^+^ and glutamate.

**Conclusion:**

The mechanism by which *B. licheniformis* regulates two macromolecules was proposed for the first time in this paper. This genetic information will facilitate the engineering of bacteria for practicable strategies for the fermentation of γ-PGA and polysaccharides for diverse applications.

**Electronic supplementary material:**

The online version of this article (doi:10.1186/s12934-017-0642-8) contains supplementary material, which is available to authorized users.

## Background

Poly-gamma-glutamic acid (γ-PGA) is a type of polyamide composed of single glutamic acids joined via γ-amide linkages between the glutamate γ-carboxyl and α-amino groups [1]. γ-PGA is a natural macromolecular polymer that is biodegradable, edible and non-toxic. Thus, γ-PGA and its derivatives have been applied in diverse fields, particularly as flocculants in water treatment and algal collection [2–4].

The selection of carbon sources for γ-PGA production is strain-dependent. Glucose and glycerol were both reported to favor γ-PGA production in most strains [5]. Importantly, glucose plays different roles in different γ-PGA-producing strains. *Bacillus licheniformis* ATCC9945a converts glucose to α-ketoglutarate via glycolysis and the TCA cycle, followed by the production of glutamic acid to synthesize γ-PGA. Moreover, glucose is reported to be a better carbon source than glycerol for the growth of *B*. *licheniformis* ATCC9945a [6]. For *Bacillus subtilis* NX-2, glucose is primarily utilized as an energy source for cell growth during γ-PGA biosynthesis, whereas glutamate in the medium is the main precursor for γ-PGA formation [7]. In contrast, using glycerol as the sole carbon source, *Bacillus amyloliquefaciens* C06 produces γ-PGA containing polysaccharides as by-products [8]. Some genetic information regarding the effect of glucose on γ-PGA synthesis has been reported. Msadek et al. demonstrated that the presence of glucose in the medium resulted in a decline in γ-PGA production because glucose suppressed the transcription of *degQ*, which activated the *CapABC* operon [9, 10].

Metabolic engineering has sought to improve γ-PGA production. In a *B. amyloliquefaciens* M306 mutant obtained by Liu et al. the γ-PGA yield increased from 3.2 to 6.8 g/L through the down-regulation of *epsD* and *yqxM* expression [11]. A study from Feng et al. demonstrated that the *epsA*-*O* deletion in *B. amyloliquefaciens* NK-1 contributed to a significant improvement in γ-PGA production (5.12 g/L), which represented a 63.2% increase compared to the wild-type strain; moreover, the γ-PGA purity improved from 76.8 to 80.4% [12]. These results indicate the existence of an unknown competition mechanism between the synthesis of γ-PGA and polysaccharides. Thus, the metabolic regulation system in these strains may control the synthesis of both extracellular polymeric substances in response to environmental changes.

In our previous studies, *B*. *licheniformis* CGMCC2876 was observed to produce extracellular polysaccharides when using glucose as the sole carbon source [13, 14], whereas poly-γ-glutamic acid (γ-PGA) was secreted when trisodium citrate and glycerol were used as the carbon sources [15]. Both of the extracellular polymeric substances exhibited high flocculating activities. Polymers with different components and molecular weights are required for different purposes, and controlling the components and molecular weights has fundamental and practical importance for commercial development [16, 17]. To better understand the metabolic mechanism underlying the section of extracellular polymeric substances, we investigated the effect of glucose on the production of polysaccharides and γ-PGA at the proteome level. Sequential window acquisition of all theoretical fragment-ion spectra (SWATH) acquisition LC–MS/MS was used to analyze the differentially expressed proteins in *B*. *licheniformis* cultured under different conditions. Finally, we proposed a mechanism for regulating the metabolism of these two macromolecules in *B*. *licheniformis*.

## Results and discussion

### *Bacillus licheniformis* cell growth in culture media with different glucose concentrations

**Fig. 1:**
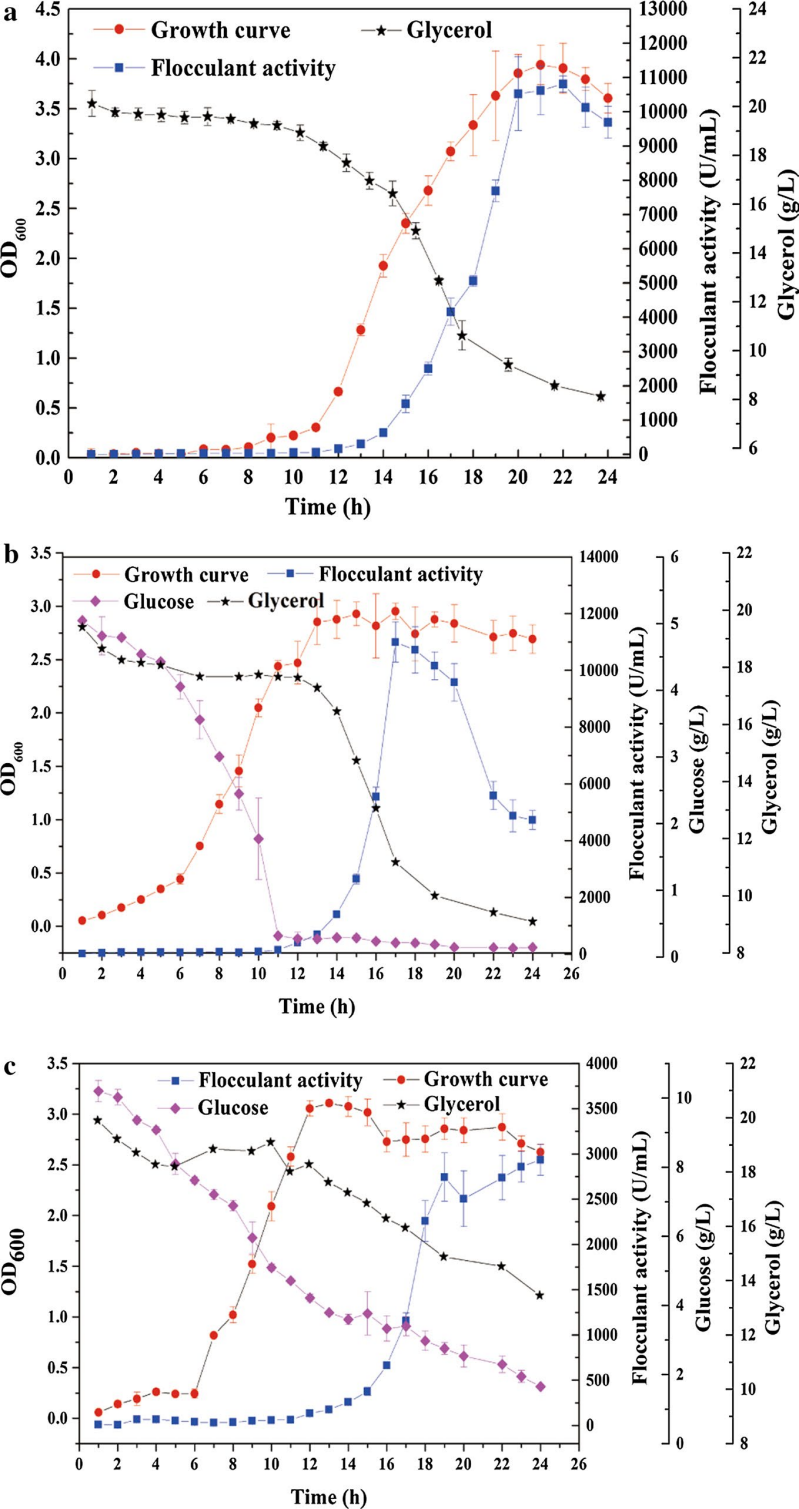
*B. licheniformis* growth curve in the three media. **a** The growth curve, glycerol consumption and flocculant activity of the fermentation culture in pure γ-PGA medium. **b** The growth curve, glycerol and glucose consumption and flocculant activity of the fermentation culture in γ-PGA medium containing 5 g/L glucose. **c** The growth curve, glycerol and glucose consumption and flocculant activity of the fermentation culture in γ-PGA medium containing 10 g/L glucose

Previous studies showed that glucose and citric acid were the better carbon sources for most γ-PGA producing strains [6, 18, 19]. Our result in Fig. 1 showed that the lag time of cell growth was significantly shortened when glucose was added and that the maximum biomass was 30% less than the biomass in the γ-PGA medium. Carbon source and C/N ratio are important factors for bacterial growth and accumulation of secondary metabolites [16]. An abundant carbon source can accelerate microbial growth. However, overabundance of carbon sources is not conducive to bacterial reproduction [20]. Additionally, glycerol was utilized after glucose was exhausted. A similar result was previously reported for *B. licheniformis* ATCC9945a, suggesting that glycerol utilization is suppressed in medium containing the glucose/glycerol mixture [6].

### Effect of glucose on *B. licheniformis* fermentation products

**Fig. 2:**
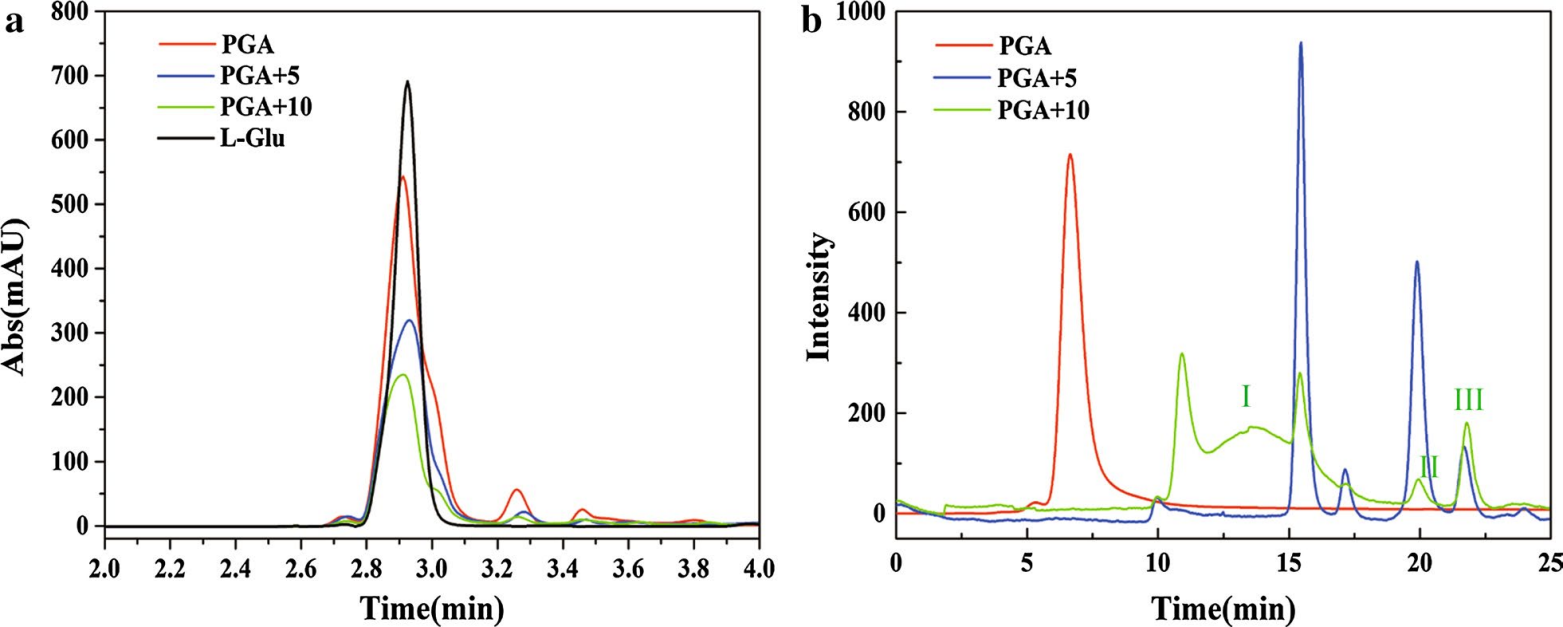
The HPLC and GPC spectra of the *B. licheniformis* fermentation products. **a** The HPLC spectra of the *B. licheniformis* hydrolyzed fermentation products under three culture media. **b** The GPC traces of the purified fermentation product in the different culture media

As shown in Table 1, 8.45 g/L polysaccharide was produced in medium containing 10 g/L glucose, which was a marked improvement from the 0.94 g/L obtained in γ-PGA medium. Our previous work demonstrated that *B. licheniformis* CGMCC2876 secreted 89% polysaccharides when glucose was supplied as the only carbon source [13, 21]. Conversely, when 5 or 10 g/L glucose was added to the γ-PGA medium, the γ-PGA concentration decreased by 31.54 and 61.62%, respectively (Fig. 2a; Table 1). Goto’s study also showed that *B. subtilis* IF03335 produced γ-PGA with polysaccharides as by-products when glucose was supplied as a co-carbon source [22]. These results suggest that glucose activates the polysaccharide synthesis pathway, which then contributes to the increase in polysaccharides secretion.

**Table 1:** The production and range of molecular mass of the fermentation products

Sample	Crude extract (g/L)	γ-PGA (g/L)	Polysaccharide (g/L)	γ-PGA (%)	Polysaccharide (%)	Mw (Da)
γ-PGA	17.988 ± 1.24	14.82 ± 1.69	0.94 ± 0.25	82.41 ± 7.39	5.2 ± 0.55	1.38 × 10^6^ ~ 2.04 × 10^7^
γ-PGA + 5 g	19.968 ± 1.57	11.27 ± 1.16	0.89 ± 0.46	56.42 ± 3.18	4.45 ± 0.79	4.25 × 10^4^; 4.57 × 10^4^ 5.38 × 10^4^; 6.38 × 10^5^
γ-PGA + 10 g	19.44 ± 1.03	6.15 ± 0.85	8.45 ± 1.02	31.63 ± 4.33	43.47 ± 6.81	2.57 ×10^4^; 3.36 × 10^4^ 5.39 × 10^5^~ 1.58 × 10^6^

### Effect of glucose on the molecular mass of the fermentation products

The molecular masses of the fermentation products are shown in Fig. 2b. In the γ-PGA medium, γ-PGA was detected with an average mass ranging from 1.38 × 10^6^ to 2.04 × 10^7^ Da. With the addition of glucose at 5 g/L, the average masses were calculated to be 4.25 × 10^4^, 4.57 × 10^4^, 5.38 × 10^4^ and 6.38 × 10^4^ Da. However, with the addition of glucose at 10 g/L, broad molecular mass distributions were observed in peak I, ranging from 5.39 × 10^5^ to 1.58 × 10^6^ Da, whereas peaks II and III showed relatively low molecular masses of 2.57 × 10^4^ and 3.36 × 10^4^ Da, respectively (Table 1). The γ-PGA hydrolase *PgdS* was directly responsible for γ-PGA degradation to regulate the molecular mass [23]. Further results showed that *pgdS* mRNA expression was up-regulated in the medium containing glucose, indicating that the addition of glucose enhanced the expression of γ-PGA hydrolase *PgdS* to decrease the molecular mass of γ-PGA (Additional file 1).

The flocculating activity of bioflocculants is closely related to both their constituents and their molecular mass [24]. Under normal circumstances, γ-PGA shows higher flocculating activity than polysaccharides at the same concentration, and the flocculating activity increases with increasing of molecular mass. In the γ-PGA medium supplemented with glucose at 10 g/L, the flocculating activity of the culture was decreased by 70% due to the 43.47% decrease in γ-PGA concentration and to the presence of smaller molecules than those secreted from γ-PGA medium (Fig. 1).

### Effect of glucose on the *B. licheniformis* metabolic pathway

**Fig. 3:**
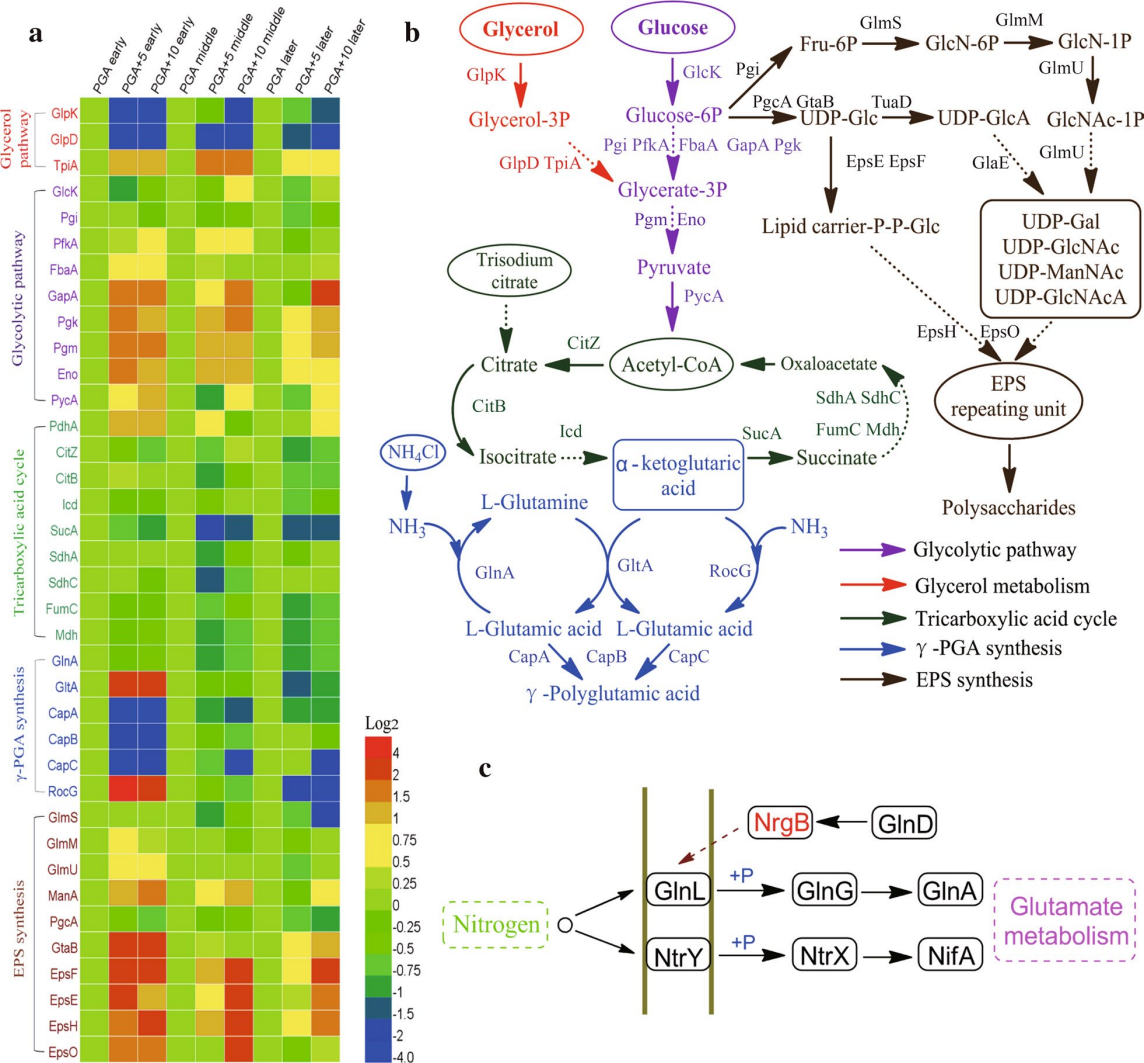
Differential proteome analyses using the SWATH acquisition LC–MS/MS method. **a** Relative protein abundances are shown here, with *red* representing highly abundant and *purple* representing the least abundant in the heat map. **b** The γ-PGA and polysaccharide de novo synthesis pathways in *B. licheniformis*. **c** The regulatory mechanism of protein *NrgB*

SWATH acquisition LC–MS/MS was employed to analyze the proteomes of *B. licheniformis* CGMCC2876 cultivated in three different media. In total, 969 intracellular proteins were detected (Additional file 2). The major proteins were involved in the EMP pathway, glycerol metabolism, and TCA cycle. Expression levels of proteins involved in the γ-PGA and polysaccharides biosynthesis are summarized and illustrated in the heat map in Fig. 3a.

#### Carbon metabolism

The activities of several enzymes involved in glycolysis increased with the addition of glucose compared with γ-PGA medium (Fig. 3a). However, the levels of these proteins decreased in the cell proteome when glucose was exhausted. Similar proteomic results were observed for *B. licheniformis* DSM 13, indicating that the expression of glycolytic proteins decreased under glucose-limiting conditions [25]. Some microbes can utilize the EMP pathway to synthesize glutamic acid as the precursor for γ-PGA synthesis [26, 27]. Similar to the findings reported in *B. subtilis* NX-2 [7], the glycolytic pathway substrates were supplied for cell growth, and the monomers were provided for polysaccharide biosynthesis (Fig. 3b).

As illustrated in Fig. 3a, glycerol metabolism was markedly inhibited by the addition of glucose, while glycerol was used as a preferred carbon source in γ-PGA synthesis, which was consistent with the reports of some other studies [6, 15, 28, 29].

TCA cycle is an essential part of γ-PGA synthetic pathways and produces a precursor (α-ketoglutaric acid) for glutamate synthesis [22, 30]. However, a large amount of pyruvic acid from glycolysis is degraded via the Krebs cycle. In the medium containing glucose at 5 g/L, the levels of *SucA* and *SdhC* decreased by 3.59- and 2.01-fold, respectively (Additional file 3) after the glucose was exhausted. The same results revealed that the decrease in *SucA* and *SdhC* was beneficial for both the accumulation of α-ketoglutaric acid and the increase in glutamate for producing γ-PGA [31]. Thus, glycerol metabolism was changed to glycolysis when glucose was added to the medium as a co-carbon source.

#### γ-PGA biosynthesis

We observed that the γ-PGA synthetic enzyme system (*CapABC*) markedly decreased during the exponential growth phase (OD_600_ = 1.0) when glucose was added (Fig. 3a). Tannler et al. reported that glucose repressed a series of secondary catabolic proteins via carbon control protein N (*CcpN*) [32]. The *cap* operon might be regulated by *CcpN*. In the medium containing glucose at 5 g/L, *CapABC* content in the cells mildly increased as glucose was exhausted, then returned to the same level as that in the cells cultured in γ-PGA medium. However, in the 10 g/L glucose medium, *CapABC* was repressed during the entire fermentation process.

Interestingly, *GltA* and *RocG*, which are both involved in γ-PGA biosynthesis, were up-regulated with the addition of glucose. *GltA* is a major regulatory link between carbon and amino acid metabolism. The lack of the *gltAB* operon limits *B. subtilis* growth on glucose/ammonium media [33]. However, *RocG* catalyzes the reaction (glutamate + NAD^+^→α-ketoglutarate + NH_3_ + NADH), which provides rapidly metabolizable carbon- or nitrogen-containing compounds for biosynthesis [34].

#### EPS biosynthesis

Several intracellular enzymes (*GlmS*, *GlmM*, *GlmU*, *EpsE*, *EpsF*, *EpsH*, *EpsO*, *ManA*, *PgcA* and *GtaB*) that participate in polysaccharide synthesis [35] were more abundant in the medium containing 10 g/L glucose than in γ-PGA medium (Fig. 3a). The expression of the *epsA*-*O* operon was maintained at a high level during polysaccharide synthesis. The bacterial cells were stimulated to activate a series of operons to synthesize polysaccharides, resulting in the diversion of the carbon flux from γ-PGA synthesis to polysaccharides.

#### Stress response proteins

Several regulatory proteins related to carbon and nitrogen metabolism are shown in Table 2. The addition of glucose resulted in an increase in *CcpA* during the exponential growth phase (OD_600_ = 1.0). *CcpA* (carbon catabolite control A) is a central regulator for coordinating the carbon metabolism and energy sources to maximize efficiency via carbon catabolite repression (CCR) and carbon catabolite activation (CCA) [36]. *CcpA* has also been reported to repress TCA cycle and activate the EMP pathway in response to the presence of glucose [37]. However, *CcpA* expression remained high when 5 g/L glucose was exhausted. Belitsky’s study showed that glutamic acid synthesis was accelerated by *CcpA* through the activation of *GltAB* and the repression of *RocG* [34]. Conversely, the deletion of *ccpA* repressed *icaADBC* transcription and inhibited polysaccharide formation in *S. epidermidis* [38]. Similarly, an *S. gordonii ccpA* mutant showed severe impairment of extracellular polysaccharide production [39].

**Table 2:** Proteomics changes in related regulatory proteins

Protein	PGA early	PGA+5 early	PGA+10 early	PGA middle	PGA+5 middle	PGA+10 middle	PGA late	PGA+5 late	PGA+10 late
NadR	1	5.93	4.804	1	1.12	1.46	1	0.86	0.94
NrgB	1	0.32	0.273	1	1.22	0.63	1	1.17	0.69
CcpA	1	1.64	1.92	1	2.13	2.34	1	0.76	0.55
CcpN	1	3.97	6.16	1	1.89	3.04	1	0.58	0.42
CodY	1	0.41	0.27	1	1.22	1.05	1	0.42	0.52

As shown in Table 2, *CcpN* increased sharply in the early growth phase when glucose was added. When glucose was exhausted, *CcpN* decreased to the level observed in the cells in γ-PGA medium. *CcpN* in *B. subtilis* has been characterized as a repressor of two gluconeogenic genes (*gapB*/*pckA*, and *glpFK*/*glpD*) that are involved in glycerol metabolism [32, 36]. When the glucose were consumed in the 5 g/L glucose medium, *CcpN* decreased to the level detected in the bacterial cells cultured in γ-PGA medium. *CcpN* remarkably altered the distribution of carbon fluxes in *B*. *licheniformis* CGMCC2876 by rerouting the main carbon fluxes from glycerol metabolism to glycolysis.

As shown in Fig. 3c, *NrgB* relayed information on the ammonium availability to downstream regulatory factors and activated *GlnA* and *GlnG*, which are involved in glutamate metabolism [40]. *NrgB* was strongly repressed during the exponential growth phase when glucose was added. Concomitant with the fermentation process, *NrgB* content in the proteome of the cells in the medium supplemented with 5 g/L glucose increased to the same level as that detected in the cells in γ-PGA medium, whereas *NrgB* levels were always low in the medium containing 10 g/L glucose. However, glutamine is an optimal nitrogen source for *B*. *licheniformis* growth. When glutamine was exhausted, alternative nitrogen sources such as ammonium were utilized [41]. In γ-PGA medium, *NrgB* facilitated ammonium utilization and activated *GlnA* and *GlnG* to promote glutamine synthesis, which was beneficial for γ-PGA synthesis. When glucose was added to the medium, the down-regulation of *NrgB* resulted in the use of glutamine as a nitrogen source and promoted cell growth. These results suggested that *NrgB* was a positive regulator of ammonium utilization and rerouted the main nitrogen flux from glutamine to ammonium.

### qPCR analysis

Nine selected genes were analyzed in detail via quantitative PCR (Table 3). These genes were selected based on their osculating roles in polysaccharide and γ-PGA synthesis with a wide range of abundances. The majority of these selected genes exhibited good correlations between the changes in mRNA expression levels and the corresponding protein abundance. *Glck* and *fruK*, which were involved in glycolysis and controlled by *CcpA*, had a decreased expression level with the addition of glucose. *Glpk mRNA expression*, which was repressed by *CcpN*, was significantly down-regulated with the addition of glucose. *GlnA*, which catalyzes the conversion of α-ketoglutarate and NH_3_ to glutamate, was mildly down-regulated at the proteome level in the presence of glucose. However, *glnA* mRNA expression was up-regulated in the cells cultured in the medium containing glucose. The *glnA* gene (glutamine synthetase) has been reported to be regulated by small regulatory RNAs (sRNAs) that base-paired with the mRNA to alter mRNA stability and translation initiation in a *Bacillus* strain [42, 43]. These results confirmed the accuracy of the proteome quantification and suggested that the regulatory processes associated with the *glnA* gene predominantly occurred at the level of post-transcriptional regulation.

**Table 3:** Transcriptomic changes in selected genes

Gene	PGA early	PGA+5 early	PGA+10 early	PGA middle	PGA+5 middle	PGA+10 middle	PGA late	PGA+5 late	PGA+10 late
*glcK*	1.00 ± 0.13	1.28 ± 0.11	3.36 ± 0.07	0.41 ± 0.06	0.06 ± 0.01	2.09 ± 0.23	0.10 ± 0.04	0.04 ± 0.01	1.09 ± 0.17
*fruK*	1.00 ± 0.21	2.46 ± 0.19	4.68 ± 0.78	1.02 ± 0.15	1.27 ± 0.28	2.02 ± 0.37	1.69 ± 0.21	0.92 ± 0.11	1.93 ± 0.40
*glpK*	1.00 ± 0.17	0.42 ± 0.05	0.08 ± 0.004	0.16 ± 0.03	0.86 ± 0.21	0.10 ± 0.02	0.21 ± 0.05	0.28 ± 0.03	0.12 ± 0.01
*icd*	1.00 ± 0.18	0.52 ± 0.09	0.48 ± 0.07	0.11 ± 0.03	0.07 ± 0.01	0.29 ± 0.04	0.05 ± 0.007	0.04 ± 0.01	0.03 ± 0.005
*glnA*	1.00 ± 0.19	1.66 ± 0.23	1.17 ± 0.19	0.67 ± 0.08	0.46 ± 0.11	0.99 ± 0.24	0.89 ± 0.17	0.33 ± 0.05	0.44 ± 0.08
*sucA*	1.00 ± 0.09	1.06 ± 0.04	0.83 ± 0.39	5.26 ± 0.57	2.59 ± 0.31	3.78 ± 0.86	0.36 ± 0.06	2.87 ± 0.33	2.71 ± 0.37
*pgsAA*	1.00 ± 0.12	0.24 ± 0.07	0.17 ± 0.02	0.37 ± 0.08	0.46 ± 0.09	0.15 ± 0.01	0.06 ± 0.007	0.07 ± 0.004	0.29 ± 0.03
*pgsB*	1.00 ± 0.07	0.13 ± 0.006	0.17 ± 0.02	0.14 ± 0.03	0.56 ± 0.02	0.05 ± 0.003	0.02 ± 0.001	0.03 ± 0.007	0.09 ± 0.01
*pgsC*	1.00 ± 0.07	0.29 ± 0.03	0.23 ± 0.01	0.03 ± 0.001	0.65 ± 0.04	0.11 ± 0.01	0.02 ± 0.005	0.01 ± 0.001	0.01 ± 0.002

**Fig. 4:**
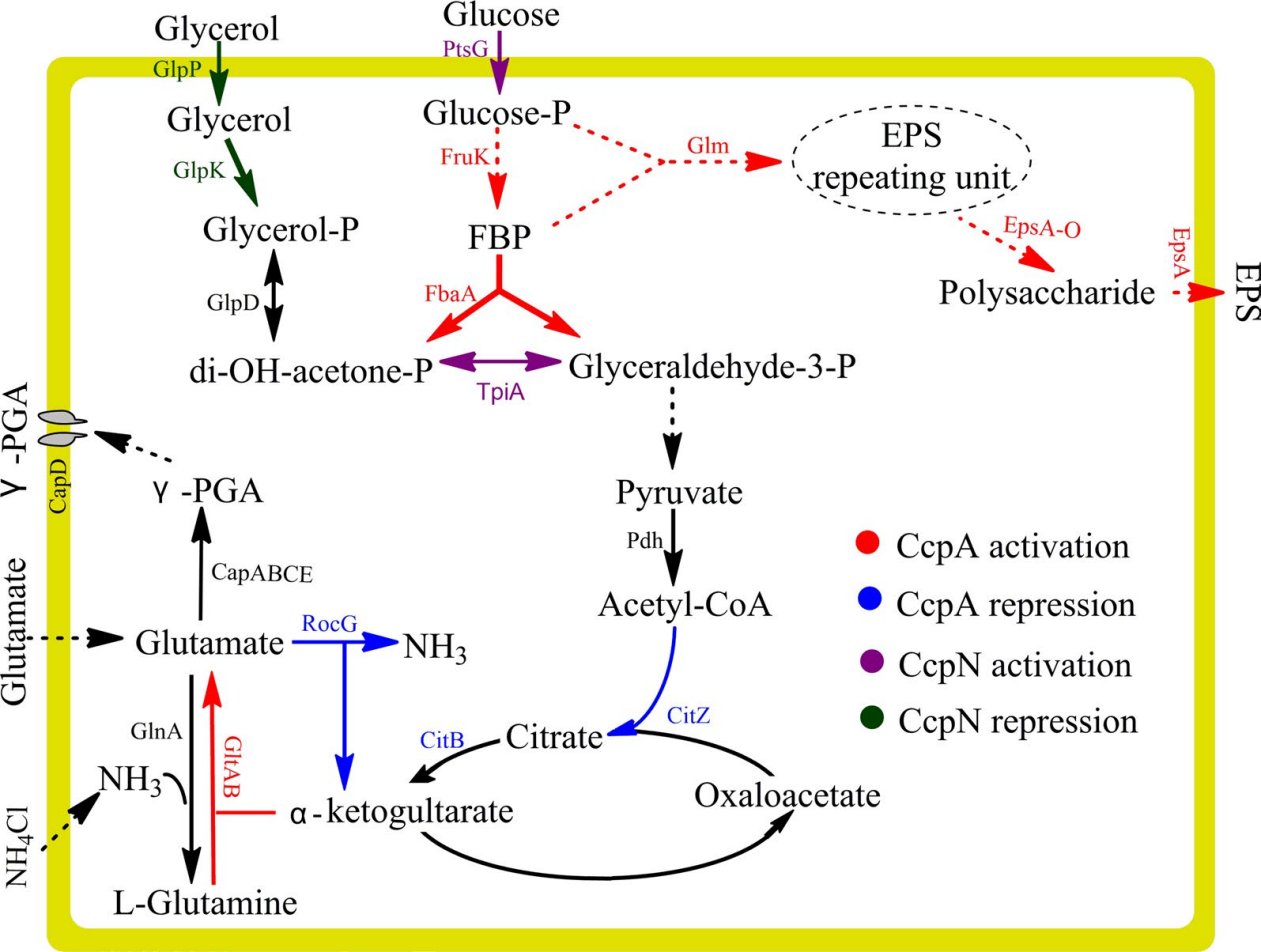
Proposed models for the central metabolic turnover process by which *B. licheniformis* produces γ-PGA and polysaccharide

Models for the carbon and nitrogen metabolic flux regulation with the effects of glucose in *B. licheniformis* CGMCC2876 are proposed in Fig. 4. When the γ-PGA medium was supplemented with glucose, the expression of the regulatory proteins (*CcpA* and *CcpN* for carbon regulation and *NrgB* for nitrogen regulation) was initiated. During the early stage of the fermentation process, *CcpA* and *CcpN* co-enhanced the glycolysis intensity and suppressed carbon flux into TCA cycle to accelerate the bacterial growth. In contrast, *CcpN* cut off the carbon flux from glycerol metabolism, further reducing γ-PGA production. *CcpA* activated a series of operons (*glm* and *epsA*-*O*) to reallocate the carbon flux and produce polysaccharide. When glucose in the medium was exhausted, the down-regulation of *CcpN* resulted in the accelerated utilization of glycerol. *CcpA* increased glutamic acid synthesis through the activation of *GltAB* and the repression of *RocG*. These changes were beneficial for γ-PGA biosynthesis. For the regulation of nitrogen metabolic flux, the down-regulation of *NrgB* switched the major source of nitrogen from NH_4_^+^ to glutamate. When glucose in the 5 g/L glucose medium was exhausted, *CcpN* expression gradually decreased, and the carbon flux from glycerol metabolism was regained. Simultaneously, when *NrgB* was up-regulated at the proteome level, the ability of the microbe to use NH_4_^+^ as its primary nitrogen source was restored, and the ability of glutamate synthesis to secrete γ-PGA was enhanced.

## Conclusion

In this study, we demonstrated the effect of glucose on fermentation products through qualitative and quantitative analyses for the first time. The γ-PGA concentration decreased by 31.54 and 61.62% when the culture medium was supplemented with glucose at 5 and 10 g/L, respectively. However, the polysaccharide concentration rose sharply from 5.2 to 43.47% with the addition of glucose at 10 g/L. The SWATH-MS method was used to clarify bacterial metabolic regulation at the protein level, resulting in the detection of 969 intracellular proteins. Carbon control proteins (*CcpA* and *CcpN*) redistributed the carbon flux from γ-PGA to polysaccharide production in the presence of glucose. Simultaneously, the regulatory protein *NrgB* converted the major nitrogen metabolic flux from NH_4_^+^ to glutamate. Overall, the central metabolic turnover processes of two different extracellular polymeric substances in *B. licheniformis* CGMCC2876 were elucidated and provided an effective fermentation strategy for regulating the production of polysaccharides and γ-PGA. Our results offer different molecular weights for diverse applications through the regulation of the ratio of the carbon source in the culture. Moreover, the regulatory mechanism provides meaningful biological information for the metabolic engineering of *B. licheniformis* for enhanced flocculate production.

## Methods

### Strain and media

The *B. licheniformis* CGMCC2876 used in this study was isolated by our laboratory [13].

The pre-culture medium consisted of the following components (g/L): glucose, 10; urea, 0.5; MgSO_4_, 0.2; KH_2_PO_4_, 0.1; K_2_HPO_4_, 0.1; NaCl, 0.1 and yeast extract, 0.5 (pH 7.2). The γ-PGA production medium contained the following components (g/L): trisodium citrate, 20; glycerol, 20; NH_4_Cl, 9; sodium glutamate, 10; MgSO_4_, 0.5; and K_2_HPO_4_, 0.5 (pH 7.2). A total of 5 or 10 g/L of glucose was added to the γ-PGA production medium.

The cells were first maintained in an Erlenmeyer flask containing pre-culture medium at 37 °C at 200 rpm for 17 h and then transferred (at a 4% inoculum ratio) into a 250 mL Erlenmeyer flask with 50 mL of γ-PGA production medium (containing 5, 10 g/L, or no glucose) for γ-PGA production.

### SWATH acquisition LC–MS/MS method analysis

For the preparation of cytoplasm proteins, the bacteria were harvested by centrifugation (12,000×*g*, 15 min, 4 °C) at three sampling times (early, middle and late). The early sampling time was during exponential growth, when OD_600_ = 1.0. The middle sampling time was 1 h after glucose was exhausted in the γ-PGA production medium supplemented with 5 g/L glucose. The late sampling time was at the end of fermentation. The pellets were washed with TE buffer (10 mM Tris and 1 mM EDTA, pH 7.5) and then resuspended in TE buffer [44]. The resuspended cells were disrupted twice at 25 kpsi at 4 °C using a homogenizer (One Shot Model, Constant Systems, UK). The cell debris was removed by centrifugation at 14,000×*g* for 15 min at 4 °C. SWATH acquisition LC–MS/MS was performed using an Eksigent nanoLC-ultra system coupled with a Triple-TOF5600 Mass Spectrometer (ABSCIEX, Canada). Details of the parameters and data analysis for SWATH were reported by Yu et al. [31].

### qPCR analysis

Concomitant with the protein extraction, RNA was immediately extracted from the samples during exponential growth (OD_600_ = 1.0), 1 h after glucose was exhausted in the γ-PGA production medium supplemented with 5 g/L glucose, and at the end of fermentation using the MiniBEST Universal RNA Extraction Kit (TaKaRa, Japan). The isolated RNA was quantified using a Spectrophotometer Q6000 (Quawell, USA). A high capacity cDNA reverse transcription kit (Applied Biosystems, USA) and a TransStart Top Green qPCR SuperMix Kit (TransGen Biotech, China) were used for reverse transcription and real-time PCR, respectively. The real-time PCR analysis was performed using a StepOne Real-Time PCR System (Applied Biosystems, USA). Reactions without the cDNA template were used as the negative controls, and γ-PGA medium without glucose was used as the reference in the calculations.

### Purification of fermentation products

After 24 h of fermentation, the culture broths were centrifuged at 10,000×*g* for 15 min to remove the cells. Three volumes of ethanol were added to the supernatant to precipitate the crude products. Then, the crude products were dissolved using distilled water and dialyzed (molecular weight cut-off of 7000 Da) in distilled water overnight. Finally, the sample was lyophilized to obtain the purified products [45].

### Qualitative and quantitative analyses of the fermentation products

The total sugar content of the purified products was determined by the phenol–sulfuric acid method using glucose as the standard solution [46]. The total protein content was measured by the Bradford method using a protein assay kit (Bio-Rad, USA).

To measure the γ-PGA content, the purified products were dissolved in 6 M HCl to hydrolyze the γ-PGA. The mixtures were maintained at 110 °C for 12 h. The hydrolysates were neutralized and metered volumetrically and then characterized by HPLC for qualitative and quantitative analysis. The HPLC analysis was performed on an Agilent 1200 HPLC system using an Agilent HC-C_18_ (25 cm × 4.6 mm) column and a UV detector (210 nm). The mobile phase consisted of 10 mM KH_2_PO_4_ (pH 2.5) and methanol (5%, v/v) at a flow rate of 0.5 mL/min. Pure sodium glutamate was used as the standard compound [47].

### Determination of the molecular masses of the fermentation products

Molecular mass was evaluated by high-performance gel permeation chromatography (HPGPC) coupled with refractive index (RI) detection using a TSK G4000PWxl column (Tosoh, Japan). The mobile phase was NaN_3_ (0.01%) at a flow rate of 0.5 mL/min. The column temperature and pressure were maintained at 30 °C and 1.3 MPa, respectively. A dextran T series (Pharmacia, Sweden) was used as the standard compound for the molecular mass determination [48].

## Additional files

**Additional file 1.** qPCR analysis of *pgdS* gene expression in *B. licheniformis*.

**Additional file 2.** Raw data of SWATH acquisition LC-MS/MS.

**Additional file 3.** Date used in Fig. 3a.

## Abbreviations

EPS: extracellular polysaccharides
γ-PGA: poly-gamma-glutamic acid
SWATH: sequential window acquisition of all theoretical fragment-ion spectra
EMP: Embden-Meyerhof-Parnas pathway
TCA: tricarboxylic acid cycle
