# Supplementary figures and images for: Effect of glucose on poly-γ-glutamic acid metabolism in Bacillus licheniformis
Source: Microb Cell Fact. 2017 Feb 8;16:22. doi: 10.1186/s12934-017-0642-8 (PMC5299652; doi:10.1186/s12934-017-0642-8)

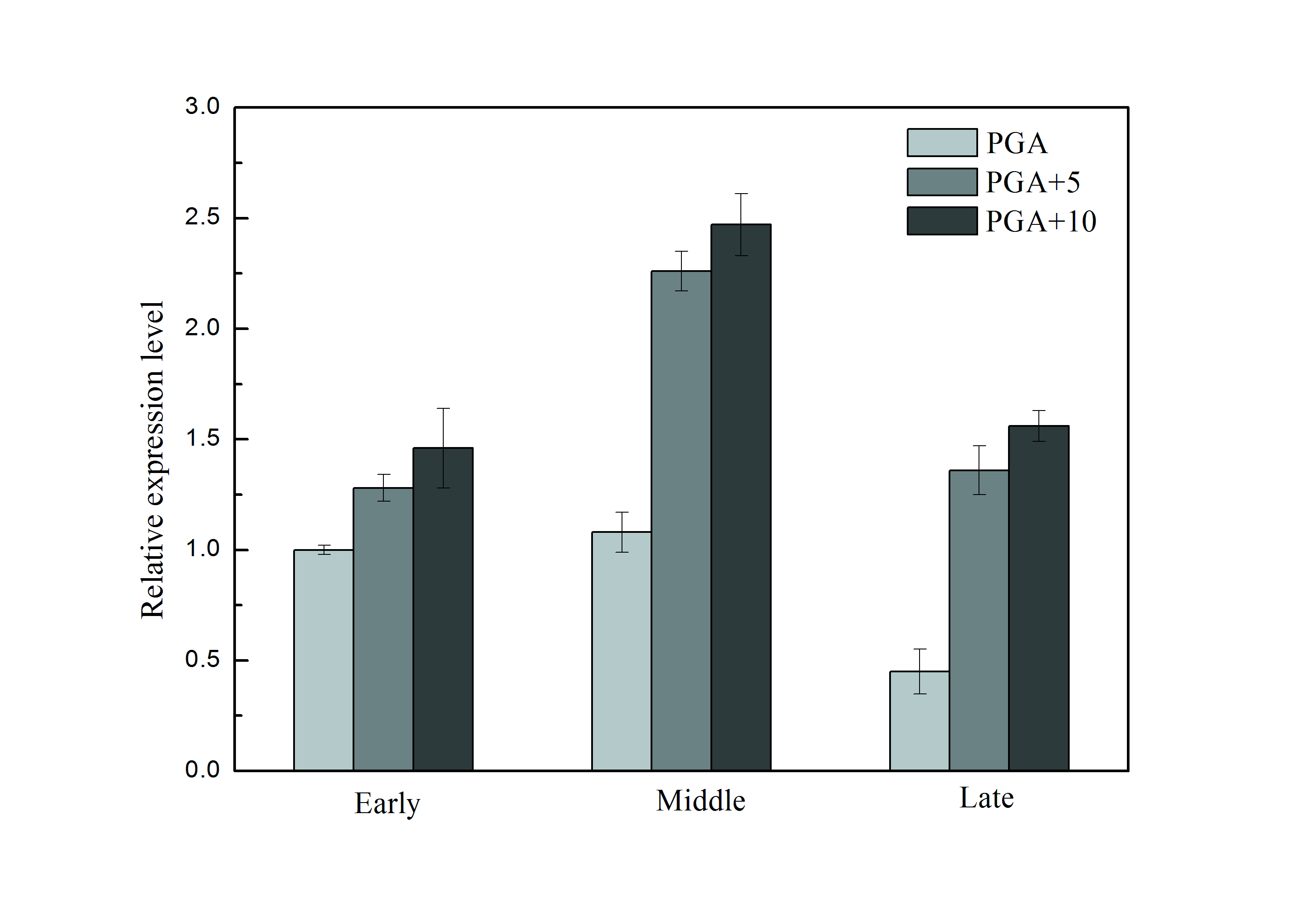

Supplement: Supplementary file 1 — Additional file 1. qPCR analysis of pgdS gene expression in B. licheniformis. [file 12934_2017_642_MOESM1_ESM.tif]
